# Assessment of clonal relationships in ipsilateral and bilateral multiple breast carcinomas by comparative genomic hybridisation and hierarchical clustering analysis

**DOI:** 10.1038/sj.bjc.6602021

**Published:** 2004-07-13

**Authors:** M R Teixeira, F R Ribeiro, L Torres, N Pandis, J A Andersen, R A Lothe, S Heim

**Affiliations:** 1 Department of Genetics, Portuguese Oncology Institute, Porto, Portugal; 2 Institute of Biomedical Sciences, University of Porto, Portugal; 3 Department of Genetics, Saint Savas Hospital, Athens, Greece; 4 Department of Pathology, Odense University Hospital, Odense, Denmark; 5 Department of Genetics, Institute for Cancer Research, The Norwegian Radium Hospital, Oslo, Norway; 6 Department of Molecular Biosciences, University of Oslo, Oslo, Norway; 7 Department of Cancer Genetics, The Norwegian Radium Hospital-University Clinic, Oslo, Norway

**Keywords:** multiple breast carcinomas, clonality, CGH, chromosome aberrations, hierarchical clustering

## Abstract

The issue of whether multiple, ipsilateral or bilateral, breast carcinomas represent multiple primary tumours or dissemination of a single carcinomatous process has been difficult to resolve, especially for individual patients. We have addressed the problem by comparative genomic hybridisation analysis of 26 tumours from 12 breast cancer patients with multiple ipsilateral and/or bilateral carcinoma lesions. Genomic imbalances were detected in 25 of the 26 (96%) tumours. Using the genomic imbalances detected in these 26 lesions as well as those previously found by us in an independent series of 35 unifocal breast carcinomas, we compared a probabilistic model for likelihood of independence with unsupervised hierarchical clustering methodologies to determine the clonal relatedness of multiple tumours in breast cancer patients. We conclude that CGH analysis of multiple breast carcinomas followed by unsupervised hierarchical clustering of the genomic imbalances is more reliable than previous criteria to determine the tumours’ clonal relationship in individual patients, that most ipsilateral breast carcinomas arise through intramammary spreading of a single breast cancer, and that most patients with bilateral breast carcinomas have two different diseases.

Breast cancer patients not rarely present multiple tumours (Lesser *et al*, 1982; Anastassiades *et al*, 1993; Vaidya *et al*, 1996). The increased emphasis in recent years on breast-sparing surgical treatment (Holland *et al*, 1985; Fisher, 1992; Connolly *et al*, 1995) has made it more clinically important to know whether multiple, ipsilateral breast carcinomas represent several primary tumours (multicentricity) or intramammary dissemination of a single carcinomatous process (multifocality). The fundamental biological interest of the issue is also profound, as it is also for bilateral breast carcinomas (Dawson *et al*, 1991; Sterns and Fletcher, 1991): are they two separate primary tumours or the metastasis from one breast to the other? Several criteria have been suggested to discriminate multiple primary tumours from metastatic lesions, including different histological growth patterns, the presence of *in situ* components in both tumours (for both ipsilateral and bilateral lesions), and localisation in different quadrants or more than 5 cm apart (for ipsilateral tumours; Dawson *et al*, 1991, 1995; Sterns and Fletcher, 1991; Dawson, 1993). However, the unspecific and quantitative nature of these criteria makes a reliable assessment of the competing hypotheses difficult in most cases.

A comparison of more fundamental biological characteristics of the different tumours could conceivably provide more reliable information as to whether they are clonally related or not. Among the principles used have been X-inactivation analysis (Noguchi *et al*, 1994a, 1994b; Shibata *et al*, 1996), comparisons of allelic imbalance patterns (Tsuda and Hirohashi, 1995; Imyanitov *et al*, 2002), or the distribution of *TP53* point mutations (Ackerman *et al*, 1995; Kinoshita *et al*, 1995; Shibata *et al*, 1996), but these studies depend heavily on statistical group comparisons and therefore are of limited value in individual patients. We have in the past successfully assessed the pathogenetic and clonal relationships among multiple breast tumours by means of chromosome banding analysis with karyotyping (Pandis *et al*, 1995; Teixeira *et al*, 1994, 1997). Since this technique provides screening information about all chromosome-level abnormalities that exist in individual cells, with each karyotypic change serving as a separate clonality marker, it is in principle highly efficient for the present purpose. However, because karyotyping is critically dependent on the successful *in vitro* culture and cytogenetic analysis of the separate cancer lesions, inadequate cell attachment, low *in vitro* mitotic index, and poor technical quality of chromosome preparations of the cells of the neoplastic parenchyma (even if for only one of the lesions) may render the case uninformative.

To circumvent the above-mentioned methodological limitations, we have used comparative genomic hybridisation (CGH), a technique that is not dependent on obtaining tumour metaphases while retaining the ability to detect copy number changes at all chromosomes at the same time, to analyse 26 tumours from 12 breast cancer patients with multiple ipsilateral and/or bilateral lesions. Furthermore, using the present data and those previously found by us in a series of 35 unifocal breast carcinomas (Teixeira *et al*, 2001), we have compared a probabilistic model for the likelihood of independence and hierarchical clustering methodologies to determine the clonal relationship among the multiple tumours in individual breast cancer patients.

## MATERIAL AND METHODS

### Tumour specimens

Samples from 26 macroscopically separate tumours arising in 12 women diagnosed with breast cancer were fresh frozen at −80°C until processed for DNA extraction. Four patients had ipsilateral tumours and another seven women had bilateral tumours. The 12th patient presented both ipsilateral and bilateral tumours (two in each breast) and had a history of radiotherapy for a mediastinal lymphoma; no iatrogenic or other predisposing factors were known for the other patients. The histopathologic classification of each tumour was made in accordance with WHO recommendations (Sobin, 1981) and was based on examination of tissue immediately adjacent to the samples processed for genetic investigation (Table 1

**Table 1:** Histopathology, genomic imbalances, and clonal relatedness of 26 ipsilateral and/or bilateral tumours from 12 breast cancer patients

**Case no.**	**Lab no.**	**Tumour**	**Diagnosis^a^**	**CGH**	***X*^b^**	**Hierarchical clustering^c^**	**Clonal relatedness^d^**
*Ipsilateral tumours*							
1	326A/92	A	COM	rev ish enh(1p22pter,1q22q24,2p23q12,3q,7p14p21, 8q21qter,9pterq33,18p11q12,18q22q23,20p11pter), dim(Xp11p21,Xp22,4p16,4q34q35,5q12q14,6p24, 6p25,10p14p15,10q23,10q24qter,11q13qter,13q14q21, 13q33q34,15q24,16q22qter)	2 × 10^−4^	C	R
	326B/92	B	DCIS	rev ish enh(3q24q29,8q21qter,9q22,20p12p13), dim(10p15,11q13q22,11q23qter,22q13)			
2	368A/92	A	DC	rev ish dim(1p34pter,8p22pter,15q13qter,16q,22q11qter)	0.26	UC	UR
	368B/92	B	DCIS+EH	rev ish enh(8p21qter,16p11pter),dim(6q14qter,8p22pter,9q22,17p12pter)			
3	426A/92	A	DC+DCIS	rev ish enh(1q,7p11p21,16p), dim(1p21pter,7q21qter,11q12q13,11q22qter,16q23qter,22q)	8 × 10^−5^	C	R
	426B/92	B	DC+DCIS	rev ish enh(1q,7p,16p),dim(1p,7q21qter,11q12q13,11q21qter,16q23qter,22q)			
4	522A/92	A	DC	rev ish enh(1q,5,8,16p11pter),dim(15q21q22,15q24q25,16q,22q)	0.002	C	R
	522B/92	B	DC	rev ish enh(1q,5,8,16p12,16p13),dim(16q)			
							
*Bilateral tumours*							
5	197/92	Right	DC	rev ish enh(1q25q32,1q41)	1	UC	UR
	198/92	Left	DC	rev ish enh(1q23q43,8p11qter,9p),dim(8p21pter,9q21qter,16q)			
6	307/92	Left	DC+DCIS	rev ish enh(1p31,1q23q42,3q26q27,6p21,6p23,8p12qter,12q15q21), dim(8p21pter,11q21qter,13q,15q21q23,15q24q26, 16q,17p12pter,17q24q25,18p11)	0.09	UC	UR
	308/92	Right	LC+LCIS	rev ish enh(8p11p21),dim(8p23,16q21qter),amp(8p12)			
7	375/92	Left	LC+LCIS	rev ish enh(8p12qter,16p),dim(9q13q31,10p12p14,11p14pter,11q22qter, 13q21qter,16q)	0.31	UC	UR
	376/92	Right	LC+LCIS	rev ish enh(Xp11p12,Xp22,Xq21qter,1q,7,14q, 15q11q25,16p11pter,20)			
8	382/92	Left	DC+DCIS	rev ish enh(1q,5pterq35,7p13p14,7p15p21,7q11q33,16p11pter),dim(16q)	0.24	UC	UR
	383/92	Right	DC+DCIS	rev ish enh(1q22q43),dim(6p22,6q21q27,11q21qter,16q,22q12q13)			
9	50/93	Left	DC+DCIS	rev ish enh(X,1q21qter,7pterq36,14q,16p,20),dim(10q)	1	UC	UR
	51/93	Right	LC+LCIS	rev ish enh(8q11q24.1), dim(2p23pter,2q21q22,6q24q27,11q24qter,13q12q13, 13q31q33,16q23qter,18q22qter)			
10	588/93	Left	DC+DCIS	rev ish enh(8p12qter,16p),dim(8p21pter,9q22,16q)	1	UC	UR
	589/93	Right	DC+DCIS	no changes			
11	257/94	Left	LC	rev ish enh(1q),dim(6q14qter,16q,17p12pter,22q)	0.001	C	R
	258/94	Right	LC	rev ish enh(1q),dim(6q14q25,16q,17p13,22q)			
							
*Ipsilateral and bilateral tumours*							
12	17/97	Right A	DC	rev ish enh(8p11p12), dim(3p21,5q31qter,6q16qter,8p22pter,13q12qter,14q32)	1 (0.37^e^)	UC	UR
	18/97	Right B	DC	rev ish enh(4q12q21)			
	19/97	Left A	DC	rev ish enh(1q21q41,15q21qter,16p11pter,17q23q25), dim(3q21q25,5q14,6q14qter,8q12q21,10q24qter, 11p,11q14qter,12p11pter,14q24,22q12qter)	0.01	UC	UR
	20/97	Left B	DC	rev ish enh(1p13p34,1q,3p24pter,4p15q35,5p15q35, 7p21,7q11qter,8,10p11pter,16p11p13,17q12q21,17q23q25,18,20,21q), dim(1p35pter,2,3p21qter,6q14q24,7p12p13,9q33qter, 10q,11p,11q23qter,13q,14q,16q21q24,17p12pter)			

a COM=comedo carcinoma; DC=ductal carcinoma; DCIS=ductal carcinoma *in situ*; LC=lobular carcinoma; LCIS=lobular carcinoma *in situ*; EH=epithelial hyperplasia.

b *X*, probability of the shared genomic imbalances occurring just by chance (see Materials and methods).

c Unsupervised hierarchical clustering analysis based on chromosome-arm coding of CGH copy number changes (see Materials and methods); C=clustered; UC=unclustered.

d Clonal relatedness based on unsupervised hierarchical clustering analysis of CGH data coded by chromosome arm; R=related; UR=unrelated.

e Calculation for comparing the two left and the two right tumours (both tumours of each side taken together).

). The study was approved by the institutional review board.

### Comparative genomic hybridisation

The CGH procedure of Kallioniemi *et al* (1994) was performed with modifications previously described by Teixeira *et al* (2001). Briefly, test (tumour) and reference (peripheral blood lymphocytes from a healthy female) DNA was extracted using standard methods and labelled in nick-translation reactions using fluorochrome-conjugated nucleotides, after which DNA fragment lengths between 300 and 2000 bp were obtained. Labelled tumour and reference DNA was mixed with unlabelled Cot-1 DNA, ethanol-precipitated, dried, and dissolved in hybridisation buffer. Normal metaphases were obtained from commercially available preparations. After denaturing the chromosomes and the DNA probe, hybridisation was allowed to occur for 2–3 days in a humidified chamber at 37°C. After a series of washes, the slides were mounted in an antifade solution with DAPI.

A total of 10 good-quality metaphase spreads were selected for analysis in each case. Three images, corresponding to each fluorochrome and the DAPI counterstain, were sequentially captured with a Cohu 4900 CCD (12-bit grey scale) camera, using an automated filter wheel coupled to a Zeiss Axioplan fluorescence microscope (Zeiss, Oberkochen, Germany) and a CytoVision system with software version 2.7 (Applied Imaging, Santa Clara, CA, USA). Chromosomes were identified based on their inverted DAPI appearance, and the relative hybridisation signal intensity was determined along each chromosome. Data obtained from the 10 cells were combined to generate average ratio profiles with 99% confidence intervals for each chromosome. A negative (normal *vs* normal) and a positive control were included in every set of experiments. In all, 10 cells of each of 10 normal *vs* normal hybridisations were used to establish the normal ratio profile with 99% confidence intervals (Kirchhoff *et al*, 1998). Copy number gains or losses were scored whenever the test and reference 99% confidence intervals did not overlap. The description of the CGH copy number changes followed the guidelines suggested by the ISCN (1995).

### Probabilistic model of clonal relatedness

In order to evaluate the clonal relatedness between any two given tumours present in the same breast cancer patient, we used the probabilistic model described by Kuukasjärvi *et al* (1997). A common clonal origin of two tumours can be inferred if they share a set of copy number gains and losses, not likely to be shared at random. In short, if *a*1, *a*2, *a*3, and so forth are specific genomic gains or losses, the probability of a particular abnormality occurring is obtained by dividing the number of occurrences of that change by the number of tumours analysed. If *T*1 is the set copy number changes of one tumour and *T*2 the set of copy number changes of another tumour, the events common to both tumours are defined by *T*1 ∩ *T*2=*c*1, *c*2, *c*3,…*ck*. Thus, a conservative estimate of the probability of a particular set of shared copy number changes occurring independently in two tumours at random is *p*(*c*1) × *p*(*c*2) × *p*(*c3*) × … × *p*(*ck*)=*X*. If two tumours share no genetic changes, *X* is by definition 1. A measure of clonal relatedness between two tumours is 1−*X*, which equals 0 if no changes are shared and approaches 1 if *X* is small. According to this model, a clonal relationship between two tumours can be inferred whenever *X*<0.05 (clonal relatedness >0.95). The probabilities of each copy number change occurring in each chromosome arm used in this probabilistic model were those previously reported by us in a series of 37 unifocal breast carcinomas (Teixeira *et al*, 2001), 35 of which were shown to harbour genomic imbalances by CGH. Copy number gains or losses at the same chromosome arm in different tumours of the same patient were considered the same only if the respective breakpoints did not differ by more than two bands.

### Hierarchical clustering of CGH data

In order to code CGH data for use with clustering software, the copy number findings were registered in an Excel spreadsheet by each of two methods: by chromosome arm (41 data points) and by chromosome band (302 data points; in both strategies excluding the p arms of the acrocentric chromosomes). The codification of copy number changes by band was carried out as follows: no change, 0; gain, +1; loss, −1; amplification (ratio >2), +2. The codification of copy number changes by chromosome arm was carried out as follows: no change, 0; gain, +1; loss, −1; amplification, +2; gain and loss from the same chromosome arm, −2. Each spreadsheet was then saved as a tab-delimited text file and loaded into the clustering software J-Express Pro (Molmine, Bergen, Norway).

Unsupervised hierarchical clustering of genomic imbalances was first performed with three pairs of primary breast carcinoma and its respective lymph node metastasis displaying different degrees of clonal relatedness (varying from 0.24–7 × 10^−9^, calculated as described above). Several software settings (including Eucledian distance *vs* Pearson correlation as distance metric and single *vs* complete linkage as cluster method) were tested to determine those that gave the best hierarchical clustering of each pair of primary tumour and lymph node metastasis (data not shown). The settings selected for the rest of the analyses were complete linkage (cluster method) and Pearson correlation (distance metric).

Besides coding the genomic imbalances separately by chromosome arm and by band, unsupervised hierarchical clustering analysis was tested as an alternative way to measure clonal relatedness among multiple breast tumours in two different ways. First, the genomic imbalances of the tumours of each breast cancer patient were compared with those of a previously reported series of 35 breast carcinomas with copy number changes (Teixeira *et al*, 2001). Second, the genomic changes of all 26 tumours of the 12 patients here reported were compared among themselves. The dendograms thus obtained group the tumours with the highest degree of genetic similarity in short-branched clusters, with longer branches indicating increasing genomic disparity. The clustering apart of tumours from the same patient, indicating that they were more similar with carcinomas of other patients than with one another, was taken as evidence of clonal unrelatedness. The clustering together of two tumours from the same woman indicated clonal relatedness among them, whereas the clustering of breast carcinomas from different women signified that they had followed similar pathogenetic pathways.

## RESULTS

### Comparative genomic hybridisation

All but one tumour presented copy number changes by CGH (Table 1). The number of imbalances per tumour ranged from 0 to 28 with an average of 8.2 per tumour. The number of copy number gains varied from 0 to 15 (average: 3.5) and the losses from 0 to 14 (average: 4.7). The chromosome arms from which material was most frequently gained were 1q (62%), 16p (46%), 8q (42%), proximal 8p (38%), and 7p (27%), whereas the ones most often displaying losses were 16q (65%), 11q (38%), 22q (35%), 6q (31%), and distal 8p and 13q (23% each). One tumour showed genomic amplification (here defined as ratio above 2.0) at 8p12.

### Clonal relatedness by the probabilistic model

The probability that the separate tumours of the 12 breast cancer patients share genomic imbalances just by chance is presented in Table 1. Whenever this probability was less than 0.05, a clonal relatedness between two tumours could be inferred with a probability greater than 0.95. With this method, a clonal relationship between the two tumours was found in three of four patients with ipsilateral breast tumours (75%) and in one of seven patients with bilateral breast carcinomas (14%). In the patient with both ipsilateral and bilateral multiple breast tumours (case 12), clonal relatedness was inferred between the two tumours in the left breast, a finding that was not confirmed by hierarchical clustering (see below).

### Hierarchical clustering of CGH data

**Figure 1:**
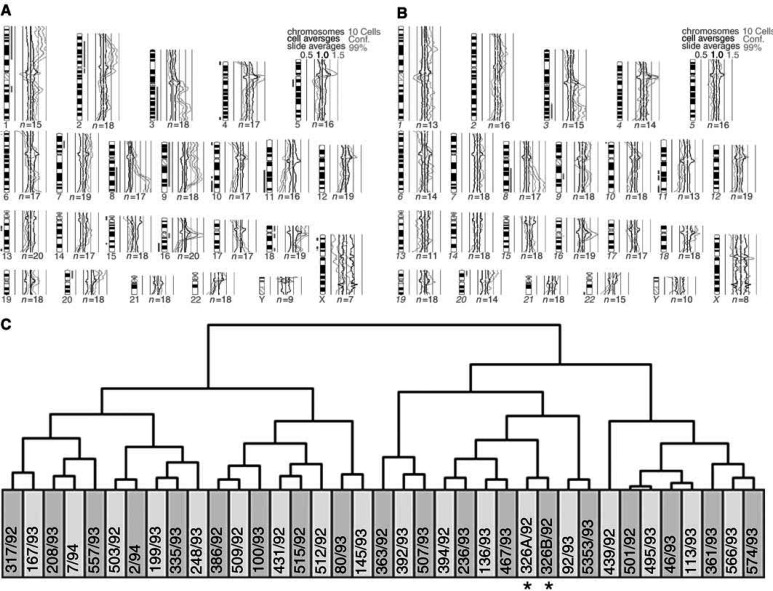
Comparative genomic hybridisation (**A**, **B**) and hierarchical clustering (**C**) of two ipsilateral breast tumours (case 1) together with 35 other breast carcinomas with copy number changes (coded by chromosome arm). Green bars to the right and red bars to the left of the chromosome ideograms indicate copy number gains and losses, respectively. See Table 1 for case numbers and a detailed description of the genomic imbalances. The two tumours cluster together (asterisks), showing that they are clonally related (multifocal breast carcinomas).

**Figure 2:**
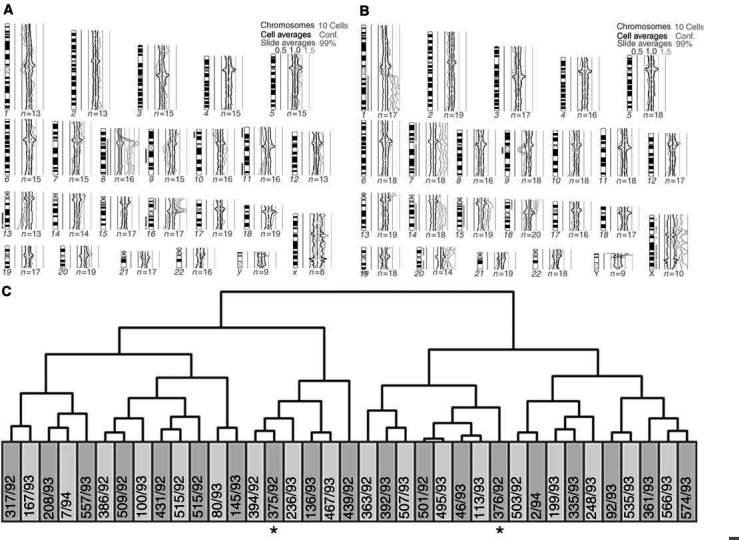
Comparative genomic hybridisation (**A**, **B**) and hierarchical clustering (**C**) of two bilateral breast carcinomas (case 7) together with other 35 breast carcinomas with copy number changes (coded by chromosome arm). Green bars to the right and red bars to the left of the chromosome ideograms indicate copy number gains and losses, respectively. See Table 1 for case numbers and a detailed description of the genomic imbalances. The two carcinomas segregate apart (asterisks), showing that they are pathogenetically more similar to tumours in other women than to one another. This is taken as indication that these bilateral carcinomas are clonally independent.

**Figure 3:**
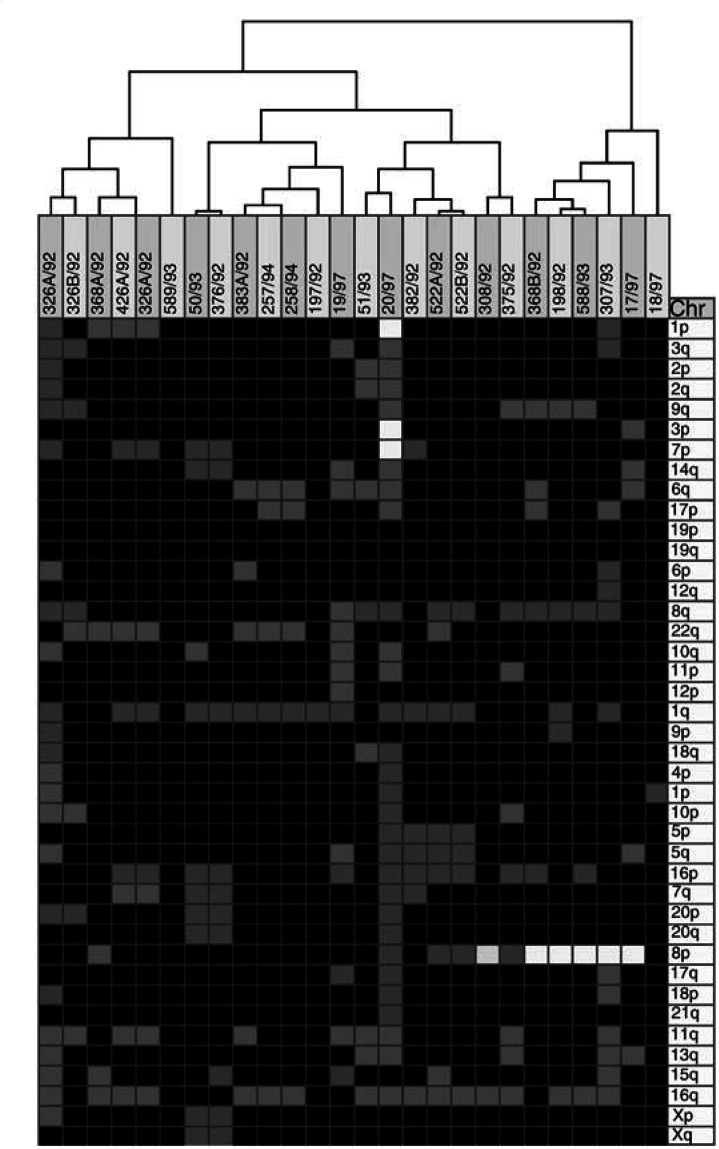
Unsupervised hierarchical clustering of all 26 tumours from the 12 breast cancer patients based on the comparative genomic hybridisation findings coded by arm. Tumour pairs clustering together represent spreading of a single disease. Tumour pairs segregating apart are independent primary carcinomas. See Table 1 for case numbers and a detailed description of the genomic imbalances.

We first used unsupervised hierarchical clustering analysis of genomic imbalances coded by chromosome arm to measure clonal relatedness among multiple breast tumours. When the copy number changes of the tumours of each breast cancer patient were compared with those of a previously reported series of 35 breast carcinomas, a clonal relationship between the two tumours was found in three of the four pairs of ipsilateral breast tumours (Figure 1

) and in one of seven pairs of bilateral breast carcinomas (Figure 2

; Table 1). The findings for these 11 patients were completely concordant with those obtained with the probabilistic model described above. This was not so for the patient with both ipsilateral and bilateral multiple breast tumours, however, as hierarchical clustering indicated that all four tumours were clonally unrelated. The clonal relationships determined by hierarchical clustering of the chromosome arm imbalances of all 26 tumours of the 12 patients here reported (Figure 3

) were in all cases similar to that obtained after comparison of each patient with the separate series of breast carcinomas.

When we used unsupervised hierarchical clustering analysis of the genomic imbalances coded by chromosome band of all 26 tumours from the 12 breast cancer patients, the clonal relationships thus determined were in all cases identical to those described above with chromosome arm coding. The same was true for all but one case when the copy number changes coded by chromosome band present in the tumours of each of the 12 patients were compared against the separate series of 35 breast carcinomas; the exception was the apparent clonal relatedness between the two left tumours of case 12.

## DISCUSSION

Comparative genomic hybridisation revealed genomic imbalances in 25 of the 26 (96%) breast tumours present in the 12 breast cancer patients, underscoring the considerable potential of the present methodology when it comes to determining the clonal relatedness among two or more tumours in the same patient. The overall pattern of copy number changes detected in this series of 26 tumours does not differ significantly from that previously found by us in a independent series of 37 breast cancer patients (Teixeira *et al*, 2001), the most frequent genomic imbalances being gain of 1q, 16p, 8q, proximal 8p, and 7p and loss of 16q, 11q, 22q, 6q, distal 8p, and 13q. Some discrepancies, such as the apparently increased frequency of 16q and 22q loss in the present series, are probably due to the fact that some of the 26 tumours were not truly independent and the differences become insignificant when only pathogenetically independent carcinomas are considered.

One way to find out whether two ipsilateral or bilateral breast tumours are two primary carcinomas or represent metastatic spreading of a single neoplastic process is to use a probabilistic model based on the frequency of genomic imbalances in an unselected series of cases (Kuukasjärvi *et al*, 1997). This model estimates the probability that shared genomic imbalances in two tumours from the same breast cancer patient occur together by chance alone. Whenever the likelihood that they occur by chance is less than 0.05, a clonal relationship between two tumours can be inferred with a probability greater than 0.95. Using these guidelines, clonal independence of the separate tumours was inferred in one of four pairs of ipsilateral breast tumours (25%) and in six of seven pairs of bilateral breast carcinomas (86%). These findings are in keeping with our previous findings using chromosome banding analysis (reviewed in Teixeira *et al*, 2002).

The probabilistic model predicts that the likelihood that the shared genetic changes of the two left tumours of patient 12 occurred by chance is only 0.01. Furthermore, in spite of formally meeting the criteria set for clonal independence, the model calculates as 0.09 the probability that the shared abnormalities of the bilateral tumours of patient 6 occurred by chance alone (i.e., the likelihood was only slightly above the 0.05 threshold). These predictions are counterintuitive when one looks at the strikingly different genomic imbalances harboured by the two lesions. This indicates that the present model may be inappropriate to determine the clonal relatedness of tumours with complex genomes, since the likelihood of detecting several shared genomic changes just by chance then increases significantly. Additionally, the probabilistic model was not able to reveal the clonal relationship between a primary breast carcinoma and its lymph node metastasis in one of three cases (data not shown), simply because they shared two of the most common genomic imbalances in breast cancer (gain of 1q and loss of 16q; *X*=0.24, i.e., not sufficient to meet the 0.05 requirement).

To overcome the above-mentioned limitations, we tested the possibility of using unsupervised hierarchical clustering of CGH data to determine more reliably when two tumours are related or clonally independent. This methodology was able to segregate correctly three pairs of primary breast carcinomas and their respective lymph node metastases with widely different genomic similarity (two of two, four of 11, and 11 of 12 copy number changes in common). The superiority of hierarchical clustering probably stems from the fact that it makes use of the entire pattern of copy number changes, not only the shared ones, present in the tumours compared. The proper clustering of primary tumours and their respective metastases took place irrespective of whether the CGH imbalances were coded by chromosome arm or by chromosome band. The only discrepancy in the results obtained by the two coding methods occurred for case 12, in which clustering of the 26 tumours by chromosome band revealed an apparent clonal relatedness between the two left tumours. This finding reveals a pitfall in the use of chromosome bands as data points, as this puts disproportionate weight on changes affecting large chromosomes compared with small ones, even if both result from single events.

Unsupervised hierarchical clustering of CGH data revealed that multiple, ipsilateral breast tumours were clonally related in three of four patients, both when the copy number changes of the tumours of each breast cancer patient were compared with those of a separate series of 35 breast carcinomas and when comparison was with the 26 tumours of this series. This finding confirms our previous conclusion based on chromosome banding data that most ipsilateral breast tumours arise through intramammary spreading of a single breast cancer (multifocal carcinomas; Teixeira *et al*, 1994, 1997). The fact that two of the three multifocal carcinomas had carcinoma *in situ* lesions in both tumours, and that these were separated by 10–40 mm of macroscopically normal-looking breast tissue, indicated that breast cancer can spread extensively via the ductal system (Ohtake *et al*, 1995; Middleton *et al*, 2002) and illustrates the limitations of these morphological criteria when it comes to discriminating multifocal from multicentric breast carcinomas (Dawson, 1993; Dawson *et al*, 1995; Tsuda and Hirohashi, 1995; Middleton *et al*, 2002). One case was classified as multicentric carcinoma by all the strategies here employed in spite of the fact that the tumours shared an 8p loss. The relatively high frequency of 8p loss in breast cancer, which makes it a marker of only poor information value, and the presence of another nine genomic changes, none of which were shared between the two tumours, leave little doubt that some ipsilateral tumours are pathogenetically independent.

The bilateral breast tumours of six of seven patients segregated apart after hierarchical clustering, indicating that they were clonally unrelated. This shows that these breast carcinomas were pathogenetically more similar to carcinomas in other women than to their contralateral tumours. Of these six patients with two genetically disparate breast carcinomas, different histological types were found in two (cases 6 and 9 had a lobular and a ductal carcinoma; three patients had two ductal carcinomas and the remaining presented two lobular carcinomas) and carcinoma *in situ* components were present in both tumours of five cases, thus supporting the conclusion that most patients with bilateral breast carcinomas have two different diseases (Dawson *et al*, 1991; Sterns and Fletcher, 1991; Pandis *et al*, 1995; Shibata *et al*, 1996; Imyanitov *et al*, 2002). This notwithstanding, one of the patients presented two bilateral lobular carcinomas sharing all five genomic imbalances by CGH, strongly arguing that one tumour must have metastasised to the contralateral breast, as we have also seen occasionally before (Pandis *et al*, 1995). We favour this interpretation over the hypothesis that a common milieu may have given rise to two clonally independent, but pathogenetically identical, breast carcinomas (Imyanitov *et al*, 2002); if this was a relevant mechanism in breast carcinogenesis, pathogenetically similar bilateral breast carcinomas should be the rule, not the exception.

We show that assessment of the clonal relationship among multiple breast tumours is significantly more informative by CGH (92% of the individual patients were successfully examined) than by chromosome banding analysis after short-term culturing (success rates of between two-thirds and three-fourths of the cases; Pandis *et al*, 1995; Teixeira *et al*, 1997). When the same cases were analysed by both techniques, discordant conclusions were obtained in a few of them (Teixeira *et al*, 1994, 1996, 1997; Pandis *et al*, 1995). Clonal selection during *in vitro* culture impairing detailed cytogenetic analysis of all tumour cell subpopulations and CGH failing to detect small clones or balanced genomic aberrations (Persson *et al*, 1999; Teixeira *et al*, 2001; Kleivi *et al*, 2002) may contribute to these discrepancies.

We conclude that CGH analysis of multiple breast carcinomas followed by unsupervised hierarchical clustering of the genomic imbalances coded by chromosome arm is more effective than previously used criteria to determine their clonal relationship in individual patients. To know precisely which ipsilateral or bilateral breast carcinomas are multiple primaries could help delineate optimal treatment strategies for these patients as well as identify families with increased susceptibility to breast cancer.
